# Serology-based subtypes of pediatric rapidly progressive glomerulonephritis and early changes in eGFR and KIM-1: a prospective cohort study

**DOI:** 10.1590/2175-8239-JBN-2026-0025en

**Published:** 2026-06-29

**Authors:** Agus Budiarto, Muhammad Riza Kurniawan, Risky Vitria Prasetyo, Ninik Asmaningsih Soemyarso, Mohammad Sjaifullah Noer

**Affiliations:** 1 Dr. Soetomo General Academic Hospital, Department of Child Health, Surabaya, Indonesia.; 2 Universitas Airlangga, Faculty of Medicine, Department of Child Health, Surabaya, Indonesia.

**Keywords:** Glomerulonephritis, Glomerular Filtration Rates, Child, Glomerular Basement Membrane, Immune Complex Diseases, Anti-Neutrophil Cytoplasmic Antibody-Associated Vasculitis

## Abstract

**Introduction:**

Pediatric rapidly progressive glomerulonephritis (RPGN) encompasses heterogeneous serology-based subtypes, including anti-glomerular basement membrane (anti-GBM), immune-complex-associated, and ANCA-associated (pauci-immune) RPGN. Although these subtypes differ in their underlying immune mechanisms, their ability to predict early renal recovery in children remains uncertain. This study aimed to evaluate the association between serology-based RPGN subtypes and early changes in renal function and tubular injury markers.

**Methods:**

This prospective cohort study included 30 children with newly diagnosed RPGN treated at Dr. Soetomo General Hospital. Patients were classified into anti-GBM, immune-complex-associated, or ANCA-associated RPGN based exclusively on serologic criteria. Estimated glomerular filtration rate (eGFR) and serum kidney injury molecule-1 (KIM-1) were measured at baseline and at 3 months after induction therapy. Primary outcomes were changes in eGFR (ΔeGFR) and serum KIM-1 (ΔKIM-1). Differences among subtypes were assessed using multivariate analysis of variance.

**Results:**

Of the 30 patients, 67% had immune-complex-associated RPGN, 20% had ANCA-associated RPGN, and 13% exhibited anti-GBM disease. The median age was 15 years. No statistically significant differences in ΔeGFR or ΔKIM-1 were observed among subtypes (MANOVA, p = 0.506), although numerical improvements varied across groups.

**Conclusion:**

Serology-based RPGN subtype was not associated with early improvement in glomerular filtration or reduction in tubular injury markers in pediatric RPGN. Early renal recovery may be influenced more by baseline disease severity and therapeutic responsiveness than by serologic subtype classification alone.

## INTRODUCTION

Rapidly progressive glomerulonephritis (RPGN) represents one of the most severe glomerular emergencies in childhood, characterized by a rapid decline in renal function, extensive crescent formation, and a substantial risk of irreversible kidney damage when treatment is delayed^1,2,3^. Despite being relatively uncommon in pediatric populations, RPGN carries a disproportionately high burden of morbidity and frequently requires aggressive immunosuppressive therapy, dialysis, or even long-term kidney replacement therapy^4,5^. Early identification of clinical and biological predictors of renal recovery remains essential to optimizing outcomes.

RPGN is commonly categorized into three major serologic and immunologic subtypes: anti-glomerular basement membrane (anti-GBM) disease, immune-complex-mediated crescentic glomerulonephritis, and pauci-immune glomerulonephritis associated with antineutrophil cytoplasmic antibodies (ANCA). These categories differ in their pathogenesis, patterns of immune deposition, and typical clinical trajectories^1,6^. Anti-GBM disease, although rare in children, is historically associated with rapid progression to kidney failure; pauci-immune RPGN similarly follows a severe course in the presence of ANCA-associated vasculitis^4^. By contrast, immune-complex RPGN—including post-infectious glomerulonephritis, lupus nephritis, IgA nephropathy, and Henoch-Schönlein purpura nephritis—is the most frequently encountered subtype in the pediatric age group^5^. Nevertheless, substantial heterogeneity exists within and between subtypes, making it difficult to predict the extent and pace of renal recovery using subtype classification alone.

Assessment of early therapeutic response in RPGN traditionally relies on estimated glomerular filtration rate (eGFR), which reflects global kidney function. However, eGFR does not directly quantify the severity of tubular injury—an important determinant of both short-term recovery and long-term prognosis. Growing interest has therefore emerged in tubular biomarkers such as kidney injury molecule-1 (KIM-1), a type I transmembrane glycoprotein markedly upregulated in injured proximal tubular epithelial cells^7^. Elevated KIM-1 concentrations have been linked to acute tubular injury, chronic tubulointerstitial damage, and progression to chronic kidney disease across various pediatric and adult populations^8,9^. Despite the potential utility of this biomarker, its role in differentiating the biological behavior of RPGN subtypes in children has not yet been clearly established.

While immunopathologic subtype has traditionally informed diagnostic categorization and selection of induction therapy, the extent to which subtype predicts short-term improvement in glomerular function (eGFR) or reduction in tubular injury (KIM-1) remains uncertain. Minimal pediatric data exist evaluating whether anti-GBM, immune-complex, or pauci-immune RPGN demonstrate distinct early recovery trajectories following standardized immunosuppressive treatment^4,5^.

The present study aims to investigate the association between serology-based RPGN subtype and early changes in eGFR and serum KIM-1 in a cohort of pediatric patients with newly diagnosed RPGN. By integrating glomerular and tubular biomarkers, this study seeks to clarify whether subtype classification provides meaningful prognostic infor­mation during the critical early phase of treatment.

## METHODS

### Study design and setting

This was a prospective observational cohort study conducted at the Pediatric Nephrology Division of Dr. Soetomo General Hospital, Surabaya, Indonesia, a tertiary referral center for pediatric renal diseases. Data were collected consecutively from children diagnosed with RPGN over the study period. All participants received standardized induction therapy and underwent follow-up assessment at 3 months. Data were collected from January to August 2025.

### Participants

Children aged 0–18 years with a newly diagnosed episode of RPGN were screened for eligibility. RPGN was defined as a rapid decline in kidney function over days to weeks accompanied by features of acute nephritic syndrome, including hematuria, proteinuria, hypertension, and varying degrees of renal impairment.

**Inclusion criteria:**

1. Confirmed diagnosis of RPGN based on clinical evaluation and serologic testing.xsxs
2. Availability of baseline eGFR and serum KIM-1 measurements.
3. Completion of 3-month follow-up laboratory testing during standardized induction therapy.

**Exclusion criteria:**

1. Pre-existing end-stage kidney disease.
2. Incomplete baseline or follow-up laboratory data.
3. Loss to follow-up before 3 months.

A total of 30 patients met the eligibility criteria and were included in the final analysis.

### Serology-based subtype classification

RPGN subtypes were classified solely based on serologic criteria, in accordance with predefined operational definitions:

- **Anti-GBM RPGN:** positive serum anti-glomerular basement membrane (anti-GBM) antibody (ELISA ≥20 RU/mL).
- **Immune-complex-associated RPGN:** hypocomple­mentemia (C3 <82 mg/dL and/or C4 <15 mg/dL), with or without antinuclear antibody (ANA) or anti-double-stranded DNA (anti-dsDNA) positivity.
- **ANCA-associated (pauci-immune) RPGN:** positive anti-myeloperoxidase (anti-MPO) and/or anti-proteinase 3 (anti-PR3) antibody (ELISA ≥20 RU/mL).

This serology-based approach reflects real-world clinical decision-making in acute pediatric RPGN, where renal biopsy may be delayed or contraindicated.

### Clinical and laboratory measurements

Baseline laboratory tests included serum creatinine, eGFR calculated using the updated Schwartz formula, blood urea nitrogen, electrolytes, complete blood count, urinalysis, complement levels (C3 and C4), anti-GBM antibodies, and ANCA (anti-PR3 and anti-MPO). Serum KIM-1 concentrations were measured using a commercially available ELISA kit according to the manufacturer’s protocol. Follow-up eGFR and KIM-1 measurements were obtained exactly 3 months after initiation of induction therapy.

Serum KIM-1 was selected for measurement due to logistical feasibility and standardized availability in our institutional laboratory during the acute phase of illness. In critically ill pediatric patients, reliable urine collection may be inconsistent, particularly in those requiring hemodialysis or intensive care support. The normal reference range for serum KIM-1 in healthy children in our laboratory is <20 pg/mL.

### Induction therapy protocol

All patients received induction therapy according to stan­dardized institutional pediatric nephrology protocols. Two treatment strategies were used.

The first regimen consisted of intravenous methylpred­nisolone pulse therapy (30 mg/kg/day, maximum 1 g/day) administered for three consecutive days, followed on day 4 by intravenous cyclophosphamide (500 mg/m^2^ body surface area, maximum 500 mg per dose). This 4-day treatment cycle (3 days of methylprednisolone followed by cyclophosphamide on day 4) was repeated every 2 weeks for a total of 6 cycles (approximately 3 months). Oral prednisone (1 mg/kg/day, maximum 60 mg/day) was administered concurrently and subsequently tapered by 5 mg per month according to clinical response.

The second regimen consisted of intravenous methyl­prednisolone pulse therapy (30 mg/kg/day, maximum 1 g/day) administered for three consecutive days every 2 weeks for 6 cycles, combined with mycophenolate mofetil (600 mg/m^2^ per dose, administered orally every 12 hours), together with oral prednisone (1 mg/kg/day, maximum 30 mg/day). Prednisone was similarly tapered by 5 mg per month based on clinical evolution. Treatment allocation was determined by clinical severity, serologic subtype, and physician judgment in accordance with institutional practice.

### Outcomes

The primary outcomes were:

1. Change in eGFR (ΔeGFR, mL/min/1.73 m^2^): calculated as 3-month eGFR minus baseline eGFR.
2. Change in serum KIM-1 (ΔKIM-1, pg/mL): calculated as 3-month KIM-1 minus baseline KIM-1.

These outcomes were compared across the three serology-based RPGN subtypes.

### Statistical analysis

Continuous variables were expressed as mean ± standard deviation or median (range) as appropriate, based on data distribution. Differences among RPGN subtypes were analyzed using multivariate analysis of variance (MANOVA) to assess the combined effect on ΔeGFR and ΔKIM-1. When the multivariate test was significant, Tukey’s honestly significant difference (HSD) *post hoc* test was planned.

In addition to Δ-based comparisons, analysis of covariance (ANCOVA) was performed using 3-month eGFR as the dependent variable and baseline eGFR as a covariate to account for potential regression-to-the-mean effects. Assumptions of ANCOVA, including homogeneity of regression slopes, were verified prior to model interpretation.

Normality of KIM-1 values was assessed using the Shapiro–Wilk test. Due to the significant deviation from normality, analyses were repeated using log-transformed KIM-1 values. Results were consistent with the primary analyses.

Given unequal group sizes, harmonic mean adjustment was used in post-hoc estimation. A two-tailed p < 0.05 was considered statistically significant. All analyses were conducted using SPSS version 21.0.

### Ethical considerations

The study received approval from the Research Ethics Committee of Dr. Soetomo General Hospital (IRB No. 1197/KEPK/XII/2024). Written informed consent was obtained from the parents or legal guardians of all participants.

## RESULTS

### Patient characteristics

Thirty pediatric patients with newly diagnosed RPGN were included in the study. The median age was 15 years (range, 3–17), with a slight predominance of female patients. According to serology-based classification, 20 patients (67%) were categorized as immune-complex-associated RPGN, 6 (20%) as ANCA-associated (pauci-immune) RPGN, and 4 (13%) as anti-GBM disease. Baseline characteristics demonstrated clear heterogeneity across subtypes. Patients with anti-GBM disease presented with more severe renal impairment, reflected by higher serum creatinine levels and a greater need for hemodialysis and ICU admission at diagnosis. In contrast, immune-complex-associated RPGN was characterized by lower complement levels and higher frequencies of ANA and anti-dsDNA positivity, whereas ANCA-associated RPGN showed preserved complement levels and ANCA subtype distribution consistent with pauci-immune disease. Baseline eGFR values did not differ significantly across subtypes (ANOVA F(2,27) = 1.314; p = 0.285). Baseline serum KIM-1 levels also did not differ significantly among subtypes (ANOVA F(2,27) = 1.631; p = 0.214).

Values are presented as mean ± standard deviation or median (range), as appropriate. The eGFR was calculated using the updated Schwartz formula. Laboratory measurements were obtained at diagnosis prior to the initiation of induction therapy. HPF: high-power field. Baseline demographic, clinical, and laboratory characteristics stratified by serology-based RPGN subtype are presented in Table 1.

**Table 1:** Baseline characteristics of patients by serology-based RPGN subtype

Variable	Immune-complex (n = 20)	ANCA-associated (n = 6)	Anti-GBM (n = 4)
**A. Demographic characteristics**			
Age, years, median (range)	14.5 (6–17)	14.5 (3–17)	15 (11–16)
Female sex, n (%)	12 (60.0)	4 (66.7)	0 (0)
Body mass index, kg/m^2^, mean ± SD	19.7 ± 0.6	20.2 ± 1.2	18.1 ± 3.3
**B. Baseline clinical characteristics**			
Systolic BP, mmHg, mean ± SD	157.1 ± 3.4	153.7 ± 7.3	151.5 ± 8.3
Diastolic BP, mmHg, mean ± SD	101.3 ± 1.2	102.0 ± 3.8	100.7 ± 4.6
Hemodialysis at presentation, n (%)	11 (55.0)	0 (0)	3 (75.0)
ICU admission, n (%)	10 (50.0)	0 (0)	3 (75.0)
**C. Baseline kidney function**			
Serum creatinine, mg/dL, median (range)	4.1 (1.8–30.4)	3.2 (1.8–4.5)	5.4 (3.2–8.2)
eGFR, mL/min/1.73 m^2^, mean ± SD	20.4 ± 16.2	30.6 ± 5.4	17.2 ± 2.3
BUN, mg/dL, mean ± SD	102.0 ± 14.2	71.5 ± 19.7	81.7 ± 23.4
**D. Urinary findings**			
Urine erythrocytes (per HPF), median (range)	24 (13–348)	38 (11–89)	31 (19–488)
Albumin-to-creatinine ratio, mg/gCr	≥300 (all patients)	≥300 (all patients)	≥300 (all patients)
**E. Disease-specific biomarkers**			
C3, mg/dL, mean ± SD	53.4 ± 4.7	111.6 ± 6.5	103.2 ± 5.1
C4, mg/dL, mean ± SD	17.1 ± 2.1	28.9 ± 5.2	28.9 ± 3.9
ANA positive, n (%)	11 (55.0)	0 (0)	0 (0)
Anti-dsDNA positive, n (%)	9 (45.0)	0 (0)	0 (0)
MPO-ANCA positive, n (%)	0 (0)	4 (66.7)	0 (0)
PR3-ANCA positive, n (%)	0 (0)	3 (50.0)	0 (0)
Anti-GBM titer, median (range)	6.9 (1.1–17.3)	8.4 (1.6–18.3)	50.6 (42.7–142.5)
**F. Induction therapy**			
Cyclophosphamide-based, n (%)	12 (60.0)	3 (50.0)	3 (75.0)
MMF-based, n (%)	8 (40.0)	3 (50.0)	1 (25.0)

### Induction therapy distribution

Induction therapy distribution varied across subtypes. Cyclophosphamide-based therapy was more frequently administered to patients with anti-GBM disease and those presenting with greater clinical severity, whereas MMF-based therapy was more commonly used in immune-complex-associated RPGN.

### Treatment-related complications

No severe opportunistic infections, mechanical ventilation due to infectious causes, or mortality occurred during the 3-month induction period. Minor infections were managed conservatively without discontinuation of immunosuppressive therapy.

### Changes in eGFR

All serology-based RPGN subtypes showed improvement in eGFR after 3 months of induction therapy. Mean ΔeGFR values were numerically highest in the anti-GBM group, followed by ANCA-associated (pauci-immune) and immune-complex-associated RPGN. However, multivariate analysis demonstrated no statistically significant difference in ΔeGFR among the three subtypes (MANOVA, p > 0.05).

Details of baseline eGFR, 3-month eGFR, and ΔeGFR for each subtype and for the overall cohort are presented in Table 2. In the overall cohort (n = 30), mean eGFR increased from 22.1 ± 15.1 to 77.3 ± 53.1 mL/min/1.73 m^2^, corresponding to a mean ΔeGFR of +55.2 ± 44.8 mL/min/1.73 m^2^.

**Table 2:** Changes in eGFR (ΔeGFR) at 3 months by RPGN subtype

RPGN subtype	Baseline eGFR (mean ± SD, mL/min/1.73 m^2^)	3-month eGFR (mean ± SD, mL/min/1.73 m^2^)	ΔeGFR (mean ± SD, mL/min/1.73 m^2^)
Anti-GBM	17.2 ± 4.6	95.5 ± 79.9	78.2 ± 82.6
Immune-complex	20.4 ± 16.2	67.1 ± 52.1	46.7 ± 39.4
ANCA-associated (pauci-immune)	30.6 ± 13.2	98.9 ± 31.9	68.3 ± 26.5
Overall cohort	22.1 ± 15.1	77.3 ± 53.1	55.2 ± 44.8

Note – Baseline and 3-month eGFR values and their corresponding changes (ΔeGFR) following induction therapy, stratified by serology-based RPGN subtype. Between-group differences were assessed using multivariate analysis of variance (MANOVA).

**Figure 1:**
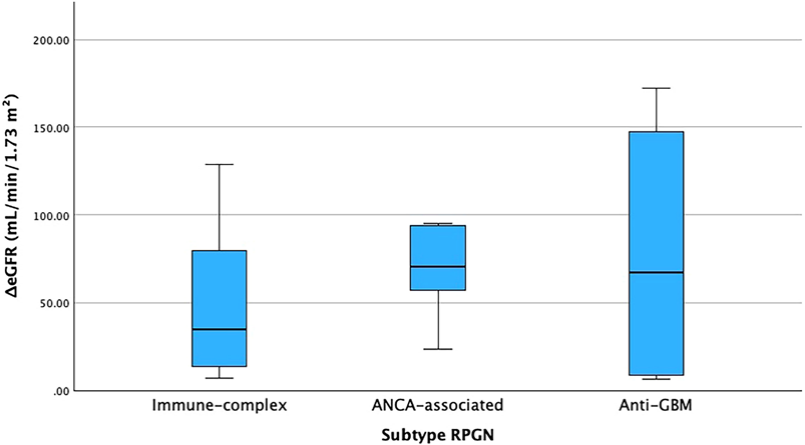
Distribution of changes in estimated glomerular filtration rate (ΔeGFR) at 3 months across serology-based RPGN subtypes.

The distribution of ΔeGFR across subtypes is illustrated in Figure 1.

### Changes in serum KIM-1

Serum KIM-1 concentrations decreased across all serology-based RPGN subtypes over the 3-month follow-up period, consistent with improvement in tubular injury. The largest numerical reduction was observed in anti-GBM RPGN, whereas the smallest reduction occurred in ANCA-associated RPGN. Nevertheless, differences in ΔKIM-1 among subtypes were not statistically significant (p > 0.05). In all groups, baseline serum KIM-1 levels were markedly elevated compared with the institutional reference range (<20 pg/mL).

Serum KIM-1 concentrations at baseline and after 3 months of induction therapy, as well as the corresponding changes (ΔKIM-1), for each serology-based RPGN subtype and for the total cohort, are presented in Table 3.

**Table 3:** Changes in serum KIM-1 (ΔKIM-1) at 3 months by RPGN subtype

RPGN subtype	Baseline KIM-1 (mean ± SD, pg/mL)	3-month KIM-1(mean ± SD, pg/mL)	ΔKIM-1(mean ± SD, pg/mL)
Anti-GBM	163.8 ± 157.5	80.7 ± 98.8	–83.1 ± 196.4
Immune-complex	138.9 ± 107.8	84.5 ± 81.9	–54.4 ± 125.3
ANCA-associated (pauci-immune)	58.7 ± 31.5	52.2 ± 86.3	–6.5 ± 87.5
Overall cohort	126.2 ± 107.6	77.5 ± 82.8	–48.6 ± 127.1

**Figure 2:**
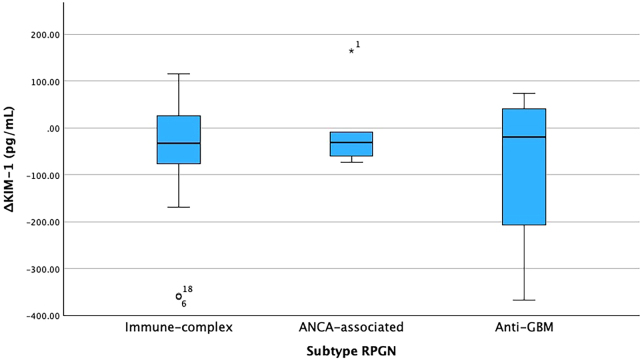
Distribution of changes in serum KIM-1 (ΔKIM-1) at 3 months across serology-based RPGN subtypes.

The distribution of ΔKIM-1 across subtypes is illustrated in Figure 2.

### ANCOVA results

To address potential regression-to-the-mean effects, ANCOVA models were performed using 3-month values as dependent variables and corresponding baseline values as covariates. After adjustment, serology-based subtype was not significantly associated with 3-month eGFR (p = 0.288). Because KIM-1 values were non-normally distributed, log-transformed ANCOVA models were considered primary for KIM-1 analysis. In the adjusted log-transformed model, subtype remained non-significant (F(2,26) = 0.202; p = 0.818; partial η^2^ = 0.015).

Because KIM-1 values were non-normally distributed (Shapiro–Wilk, p < 0.001), ANCOVA analyses were repeated using log-transformed values. After transformation, serology-based subtype remained non-significant (F(2,26) = 0.202; p = 0.818; partial η^2^ = 0.015), confirming the robustness of the findings. No significant interaction between baseline values and subtype was observed, supporting the assumption of homogeneity of regression slopes. The results of the ANCOVA analysis evaluating 3-month outcomes after adjustment for baseline values are summarized in Table 4.

**Table 4:** ANCOVA model for 3-month eGFR adjusted for baseline eGFR

Outcome	Covariate F	Covariate p	Subtype F	Subtype p	R^2^
3-month eGFR	20.141	<0.001	1.307	0.288	0.479
3-month KIM-1*	0.196	0.662	0.202	0.818	0.032

Note – *KIM-1 values were log-transformed for ANCOVA analysis.

### Multivariate analysis

Multivariate analysis of variance combining ΔeGFR and ΔKIM-1 demonstrated no significant overall effect of serology-based RPGN subtype (Wilks’ Lambda = 0.882; F(4,52) = 0.840; p = 0.506; partial η^2^ = 0.061). These findings indicate that subtype classification was not associated with combined short-term changes in glomerular and tubular markers. Given the small subgroup sizes, particularly in the anti-GBM group (n = 4), the study may be underpowered to detect moderate effect sizes; therefore, non-significant findings should not be interpreted as evidence of equivalence.

### Summary of key findings

- Serology-based RPGN subtype was not associated with short-term improvement in eGFR.
- Serology-based RPGN subtype was not associated with a reduction in serum KIM-1.
- Observed differences across subtypes were numerical and did not reach statistical significance.
- Early renal recovery appeared to be influenced more by baseline disease severity and therapeutic response than by serologic subtype classification.

## DISCUSSION

This study evaluated whether serology-based subtypes of pediatric RPGN, namely anti-glomerular basement membrane (anti-GBM) disease, immune-complex-associated RPGN, and ANCA-associated (pauci-immune) RPGN, were linked to short-term renal recovery following induction therapy. Renal recovery was assessed by changes in estimated glomerular filtration rate (ΔeGFR) and serum kidney injury molecule-1 (ΔKIM-1). The principal finding of this study is that serology-based RPGN subtype was not significantly associated with early improvement in either glomerular function or tubular injury markers during the first 3 months of treatment. These findings underscore that, in pediatric RPGN, early functional recovery may not directly reflect etiologic differences captured by serologic classification during the acute treatment phase.

### Serologic diversity but similar early recovery patterns

RPGN represents a heterogeneous clinical syndrome characterized by rapid loss of kidney function over a short period of time, traditionally categorized according to distinct immunologic mechanisms^1^. Anti-GBM disease is mediated by autoantibodies directed against the α3 chain of type IV collagen, leading to aggressive glomerular injury^2,3^. ANCA-associated RPGN is characterized by necrotizing crescentic glomerulonephritis with minimal immune deposition and systemic vasculitis features^10,11^. Immune-complex-associated RPGN encompasses a broad spectrum of disorders, including post-infectious glomerulonephritis, IgA nephropathy, and lupus nephritis, which often demonstrate variable and potentially reversible courses in children^12^.

Despite these biologically distinct upstream mechanisms, our study demonstrated no statistically significant differences in ΔeGFR or ΔKIM-1 across serology-based RPGN subtypes during the early treatment phase. This finding suggests that, in the acute setting, early renal recovery in pediatric RPGN may be governed more by shared downstream inflammatory pathways and responsiveness to immunosuppressive therapy than by the initiating serologic process itself. Such convergence of early recovery trajectories may obscure subtype-specific differences that could become apparent only with longer follow-up.

It is plausible that, despite different initiating immune triggers, the final common pathway of crescent formation and acute glomerular inflammation converges toward similar short-term functional trajectories once immunosuppressive therapy is initiated. This biological convergence may attenuate early inter-subtype differences in measurable renal function.

### Interpretation of eGFR findings

Although anti-GBM RPGN showed the largest numerical increase in eGFR, followed by ANCA-associated and immune-complex-associated RPGN, these differences did not reach statistical significance. Several factors may explain this observation. First, induction therapy in RPGN is designed to rapidly suppress glomerular inflammation irrespective of etiology, potentially minimizing early inter-subtype differences in functional recovery^1^. Second, baseline disease severity appeared to influence ΔeGFR; patients presenting with more severe renal impairment may demonstrate larger numerical improvements due to a greater margin for recovery, without necessarily achieving superior long-term outcomes^2^. Third, the unequal distribution of patients across subtypes and the limited sample size reduced statistical power to detect modest between-group differences.

A 3-month follow-up primarily captures early inflammatory response rather than long-term structural remodeling. Subtype-related differences in fibrosis progression, relapse risk, or chronic kidney disease development may only emerge over longer observation periods^3^.

### Interpretation of KIM-1 findings

KIM-1 is a well-established biomarker of proximal tubular injury, markedly upregulated following epithelial damage and reflective of both acute and chronic tubular stress^13,14^. In the present study, serum KIM-1 levels declined across all serology-based RPGN subtypes after induction therapy, consistent with improvement in tubular injury. However, the magnitude of ΔKIM-1 did not differ significantly among subtypes.

This finding likely reflects the slower and more heterogeneous kinetics of tubular repair compared with glomerular functional recovery. Tubular epithelial regeneration often lags behind improvements in filtration, and early changes in KIM-1 may therefore be less sensitive to etiologic differences in the acute phase^13^. In addition, substantial inter-individual variability in baseline KIM-1 levels and changes over time further limits the discriminatory capacity of short-term KIM-1 dynamics across serologic subtypes. These observations suggest that longer follow-up may be required to determine whether tubular biomarkers can meaningfully differentiate recovery trajectories in pediatric RPGN.

Because Δ-based analyses may be influenced by baseline severity, ANCOVA models were performed to adjust for baseline values. Even after controlling for baseline eGFR and KIM-1, serology-based subtype was not associated with short-term renal recovery. These findings strengthen the robustness of the primary results and suggest that early functional improvement may be independent of serologic classification.

Although urinary KIM-1 is more commonly used as a biomarker of proximal tubular injury, serum KIM-1 was measured in this study for practical and feasibility reasons in an acute pediatric setting. Urinary collection may be unreliable in critically ill patients, particularly those undergoing hemodialysis. However, serum KIM-1 may be influenced by systemic inflammation, reduced renal clearance, and circulating protein dynamics, which may limit its specificity compared with urinary measurements. This represents an important limitation when interpreting tubular injury dynamics in our cohort.

### Clinical implications

The findings of this study have important clinical implications. While serologic classification remains essential for diagnostic orientation and therapeutic decision-making in pediatric RPGN, it should not be used in isolation to predict short-term renal recovery. The absence of significant differences in early changes in eGFR and KIM-1 among serology-based subtypes indicates that early prognosis is not subtype-dependent. Instead, timely diagnosis and prompt initiation of appropriate immunosuppressive therapy are likely to play a more decisive role in determining early outcomes than serologic subtype alone^6^. Clinically, these findings suggest that early management strategies in pediatric RPGN should prioritize disease severity and timely initiation of immunosuppressive therapy rather than rely on serologic subtype alone for short-term prognostication.

### Strengths, limitations, and future directions

The relatively small sample size, particularly in the anti-GBM (n = 4) and ANCA-associated (n = 6) subgroups, substantially limits statistical power and increases the risk of type II error. Therefore, the absence of statistically significant differences should not be interpreted as evidence of equivalence across serology-based subtypes. Given the rarity of pediatric RPGN, this study should be considered exploratory, and largermulticenter cohorts are needed to confirm these findings. To our knowledge, this is one of the few prospective pediatric studies evaluating both glomerular function and tubular injury markers simultaneously in early RPGN. The inclusion of ANCOVA modeling and log-transformed analyses strengthens the robustness of the findings despite the limited sample size.

Nevertheless, several limitations must be acknowledged. The small sample size, particularly in the anti-GBM and ANCA-associated groups, limited statistical power. The relatively short follow-up period may not capture long-term renal outcomes or progression to chronic kidney disease. Because subtype classification was based exclusively on serologic criteria, misclassification bias cannot be excluded, particularly within the immune-complex group, which may include heterogeneous entities such as lupus nephritis or infection-related glomerulonephritis. Histopathologic confirmation could have allowed assessment of crescent percentage, chronicity index, and tubulointerstitial fibrosis, which are known predictors of renal outcome. The absence of these structural parameters limits our ability to correlate biological subtype with tissue-level severity^15^. Future studies should involve larger, multicenter pediatric cohorts with longer follow-up durations to better delineate subtype-specific recovery trajectories. Integration of additional tubular biomarkers and structural assessments may further clarify the relationship between serologic phenotype, tissue injury, and long-term renal outcomes^16^. In conclusion, our findings suggest that early renal functional recovery in pediatric RPGN may reflect shared inflammatory dynamics rather than serologic categorization alone, underscoring the importance of timely therapeutic intervention across subtypes.

## Data Availability

Data are available from the corresponding author upon reasonable request, with patient anonymity maintained.
