# Supplementary material for: Serology-based subtypes of pediatric rapidly progressive glomerulonephritis and early changes in eGFR and KIM-1: a prospective cohort study
Source: J Bras Nefrol. 2026 Jun 29;48(4):e20260025. doi: 10.1590/2175-8239-JBN-2026-0025en (PMC13395474; doi:10.1590/2175-8239-JBN-2026-0025en)
Supplement: Translation

# Translation: Subtipos sorológicos da glomerulonefrite rapidamente progressiva pediátrica e alterações precoces na TFGe e na KIM-1: um estudo de coorte prospectivo

**DOI:** 10.1590/2175-8239-JBN-2026-0025pt

**Authors:** Agus Budiarto, Muhammad Riza Kurniawan, Risky Vitria Prasetyo, Ninik Asmaningsih Soemyarso, Mohammad Sjaifullah Noer

**Affiliations:** 1 Dr. Soetomo General Academic Hospital, Department of Child Health, Surabaya, Indonesia.; 2 Universitas Airlangga, Faculty of Medicine, Department of Child Health, Surabaya, Indonesia.

**Keywords:** Glomerulonefrite, Taxa de Filtração Glomerular, Pediátrica, Membrana Basal Glomerular, Doenças do Complexo Imunitário, Vasculite Associada a Anticorpo Anticitoplasma de Neutrófilos

## Abstract

**Introdução:**

A glomerulonefrite rapidamente progressiva (GNRP) pediátrica compreende subtipos heterogêneos baseados em sorologia, incluindo doença anti-membrana basal glomerular (anti-GBM), associada a imunocomplexos e associada a ANCA (pauci-imune). Embora apresentem mecanismos imunológicos distintos, sua capacidade de predizer a recuperação renal precoce em crianças permanece incerta. Este estudo avaliou a associação entre subtipos sorológicos de GNRP e alterações precoces em marcadores de função renal e lesão tubular.

**Métodos:**

Estudo de coorte prospectivo com 30 crianças com diagnóstico recente de GNRP, tratadas no *Dr. Soetomo General Hospital*. Os pacientes foram classificados em GNRP anti-GBM, associada a imunocomplexos ou associada a ANCA, com base exclusivamente em critérios sorológicos. A taxa de filtração glomerular estimada (TFGe) e a KIM-1 sérica foram medidas no início do estudo e após 3 meses de terapia de indução. Os desfechos primários foram as variações da TFGe (ΔTFGe) e da KIM-1 (ΔKIM-1). Diferenças entre os subtipos foram avaliadas por análise multivariada de variância.

**Resultados:**

Dos 30 pacientes, 67% apresentaram GNRP associada a imunocomplexos, 20% associada a ANCA e 13% apresentaram doença anti-GBM. A idade mediana foi de 15 anos. Não houve diferenças estatisticamente significativas em ΔTFGe ou ΔKIM-1 entre os subtipos (MANOVA, p = 0,506), embora melhorias numéricas tenham variado entre os grupos.

**Conclusão:**

O subtipo sorológico de GNRP não esteve associado à melhora precoce da filtração glomerular nem à redução de marcadores de lesão tubular. A recuperação renal precoce pode ser mais influenciada pela gravidade inicial da doença e pela resposta terapêutica do que pela classificação sorológica isoladamente.

## INTRODUÇÃO

A glomerulonefrite rapidamente progressiva (GNRP) representa uma das emergências glomerulares mais graves na infância, caracterizada por um rápido declínio da função renal, extensa formação de crescentes glomerulares e por um risco substancial de dano renal irreversível quando o tratamento é retardado^1,2,3^. Apesar de ser relativamente incomum na população pediátrica, a GNRP acarreta uma carga desproporcionalmente elevada de morbidade e frequentemente requer terapia imunossupressora agressiva, diálise ou até mesmo terapia renal substitutiva a longo prazo^4,5^. A identificação precoce de preditores clínicos e biológicos de recuperação renal permanece essencial para a otimização dos desfechos clínicos.

A GNRP é comumente categorizada em três principais subtipos sorológicos e imunológicos: doença por anticorpos anti-membrana basal glomerular (anti-GBM), glomerulonefrite crescêntica mediada por imunocomplexos e glomerulonefrite pauci-imune associada a anticorpos anticitoplasma de neutrófilos (ANCA). Essas categorias diferem quanto à patogênese, aos padrões de deposição imunológica e às trajetórias clínicas típicas^1,6^. A doença anti-GBM, embora rara em crianças, está historicamente associada à rápida progressão para insuficiência renal; de forma semelhante, a GNRP pauci-imune pode evoluir com curso grave na presença de vasculite associada a ANCA^4^. Em contraste, a GNRP mediada por imunocomplexos — incluindo glomerulonefrite pós-infecciosa, nefrite lúpica, nefropatia por IgA e nefrite da púrpura de *Henoch–Schönlein* — constitui o subtipo mais frequentemente encontrado na população pediátrica^5^. No entanto, existe considerável heterogeneidade tanto dentro quanto entre esses subtipos, o que dificulta a previsão da extensão e da velocidade da recuperação renal quando se utiliza apenas a classificação por subtipos.

A avaliação da resposta terapêutica precoce na GNRP tradicionalmente baseia-se na taxa de filtração glomerular estimada (TFGe), que reflete a função renal global. No entanto, a TFGe não quantifica diretamente a gravidade da lesão tubular — um determinante importante tanto da recuperação em curto prazo quanto do prognóstico em longo prazo. Consequentemente, tem havido crescente interesse em biomarcadores tubulares, como a molécula-1 de lesão renal (KIM-1), uma glicoproteína transmembrana do tipo I significativamente superexpressa nas células epiteliais lesionadas do túbulo proximal^7^. Concentrações elevadas de KIM-1 têm sido associadas à lesão tubular aguda, ao dano túbulo-intersticial crônico e à progressão para doença renal crônica em diversas populações pediátricas e adultas^8,9^. Apesar do potencial valor desse biomarcador, seu papel na diferenciação do comportamento biológico dos subtipos de GNRP em crianças ainda não foi claramente estabelecido.

Embora o subtipo imunopatológico tenha tradicionalmente orientado a categorização diagnóstica e a escolha da terapia de indução, permanece incerto até que ponto esse subtipo é capaz de predizer a melhora em curto prazo da função glomerular (TFGe) ou a redução da lesão tubular (KIM-1). Existem poucos dados pediátricos avaliando se a GNRP do tipo anti-GBM, mediada por imunocomplexos ou pauci-imune, apresenta trajetórias distintas de recuperação precoce após tratamento imunossupressor padronizado^4,5^.

O presente estudo tem como objetivo investigar a associação entre o subtipo de GNRP baseado em sorologia e as alterações precoces na TFGe e nos níveis séricos de KIM-1 em uma coorte de pacientes pediátricos com diagnóstico recente de GNRP. Ao integrar biomarcadores glomerulares e tubulares, este estudo busca esclarecer se a classificação por subtipo fornece informações prognósticas relevantes durante a fase inicial crítica do tratamento.

## MÉTODOS

### Desenho e local do estudo

Este estudo consistiu em uma coorte observacional prospectiva conduzida na Divisão de Nefrologia Pediátrica do Hospital Geral Dr. Soetomo, em Surabaya, Indonésia, um centro terciário de referência para doenças renais pediátricas. Os dados foram coletados consecutivamente de crianças diagnosticadas com GNRP durante o período da análise. Todos os participantes receberam terapia de indução padronizada, sendo submetidos à avaliação de seguimento após 3 meses. A coleta de dados foi realizada entre janeiro e agosto de 2025.

### Participantes

Crianças com idade entre 0 e 18 anos com um episódio recém-diagnosticado de GNRP foram avaliadas quanto à elegibilidade. A GNRP foi definida como um rápido declínio da função renal ao longo de dias a semanas, acompanhado por características de síndrome nefrítica aguda, incluindo hematúria, proteinúria, hipertensão e graus variáveis de comprometimento renal.

**Critérios de inclusão:**

1. Diagnóstico confirmado de GNRP com base na avaliação clínica e em testes sorológicos.
2. Disponibilidade de medições basais de TFGe e de KIM-1 sérica.
3. Conclusão da avaliação laboratorial de seguimento após 3 meses durante a terapia de indução padronizada.

**Critérios de exclusão:**

1. Doença renal em estágio terminal pré-existente.
2. Dados laboratoriais basais ou de seguimento incompletos.
3. Perda de seguimento antes de 3 meses.

Um total de 30 pacientes preencheu os critérios de elegibilidade e foi incluído na análise final.

### Classificação dos subtipos baseada em sorologia

Os subtipos de GNRP foram classificados exclusivamente com base em critérios sorológicos, de acordo com definições operacionais previamente estabelecidas:

- **GNRP anti-GBM:** anticorpo sérico anti-membrana basal glomerular (anti-GBM) positivo (ELISA ≥20 RU/mL).
- **GNRP associada a imunocomplexos:** hipocomple­mentemia (C3 <82 mg/dL e/ou C4 <15 mg/dL), com ou sem positividade para anticorpo antinuclear (ANA) ou anticorpo anti-DNA de dupla hélice (anti-dsDNA).
- **GNRP associada a ANCA (pauci-imune):** anticorpo anti-mieloperoxidase (anti-MPO) e/ou anti-proteinase 3 (anti-PR3) positivo (ELISA ≥20 RU/mL).

Essa abordagem baseada em sorologia reflete a tomada de decisão clínica no mundo real na GNRP pediátrica aguda, na qual a biópsia renal pode ser adiada ou contraindicada.

### Medições clínicas e laboratoriais

Os exames laboratoriais basais incluíram creatinina sérica, TFGe calculada pela fórmula de *Schwartz* atualizada, nitrogênio ureico sanguíneo, eletrólitos, hemograma completo, urinálise, níveis de complemento (C3 e C4), anticorpos anti-GBM e ANCA (anti-PR3 e anti-MPO). As concentrações séricas de KIM-1 foram medidas utilizando um kit ELISA comercialmente disponível, de acordo com o protocolo do fabricante. As medições de TFGe e KIM-1 de seguimento foram obtidas exatamente 3 meses após o início da terapia de indução.

A KIM-1 sérica foi selecionada para medição devido à viabilidade logística e à disponibilidade padronizada em nosso laboratório institucional durante a fase aguda da doença. Em pacientes pediátricos criticamente enfermos, a coleta confiável de urina pode ser inconsistente, particularmente naqueles que necessitam de hemodiálise ou de suporte em unidade de terapia intensiva. O intervalo de referência normal para KIM-1 sérica em crianças saudáveis em nosso laboratório é <20 pg/mL.

### Protocolo de terapia de indução

Todos os pacientes receberam terapia de indução de acordo com protocolos padronizados institucionais da nefrologia pediátrica. Duas estratégias terapêuticas foram utilizadas.

O primeiro regime consistiu em pulsoterapia intravenosa com metilprednisolona (30 mg/kg/dia, dose máxima de 1 g/dia) administrada por três dias consecutivos, seguida, no dia 4, por ciclofosfamida intravenosa (500 mg/m^2^ de superfície corporal, dose máxima de 500 mg por aplicação). Esse ciclo terapêutico de 4 dias (3 dias de metilprednisolona seguidos de ciclofosfamida no dia 4) foi repetido a cada 2 semanas, totalizando 6 ciclos (aproximadamente 3 meses). Prednisona oral (1 mg/kg/dia, dose máxima de 60 mg/dia) foi administrada concomitantemente, com redução gradual subsequente de 5 mg por mês, de acordo com a resposta clínica.

O segundo regime consistiu em pulsoterapia intravenosa com metilprednisolona (30 mg/kg/dia, dose máxima de 1 g/dia), administrada por três dias consecutivos a cada 2 semanas, por um total de 6 ciclos, combinada com micofenolato mofetil (600 mg/m^2^ por dose, administrado por via oral a cada 12 horas), juntamente com prednisona oral (1 mg/kg/dia, dose máxima de 30 mg/dia). A prednisona foi igualmente reduzida gradualmente em 5 mg por mês, com base na evolução clínica. A alocação do tratamento foi determinada pela gravidade clínica, pelo subtipo sorológico e pelo julgamento médico, de acordo com a prática institucional.

### Desfechos

Os desfechos primários foram:

1. Variação da TFGe (ΔTFGe, mL/min/1,73 m^2^): calculada como a TFGe após 3 meses menos a TFGe basal.
2. Variação da KIM-1 sérica (ΔKIM-1, pg/mL): calculada como o valor da KIM-1 após 3 meses menos o valor basal de KIM-1.

Esses desfechos foram comparados entre os três subtipos de GNRP baseados em sorologia.

### Análise estatística

As variáveis contínuas foram expressas como média ± desvio padrão ou mediana (intervalo), conforme apropriado, de acordo com a distribuição dos dados. As diferenças entre os subtipos de GNRP foram analisadas por meio de análise multivariada de variância (MANOVA) para avaliar o efeito combinado sobre ΔTFGe e ΔKIM-1. Quando o teste multivariado foi significativo, planejou-se o teste *post hoc* de *Tukey (honestly significant difference* [HSD]).

Além das comparações baseadas em Δ, foi realizada análise de covariância (ANCOVA), utilizando a TFGe aos 3 meses como variável dependente e a TFGe basal como covariável, a fim de controlar possíveis efeitos de regressão à média. As premissas da ANCOVA, incluindo a homogeneidade das inclinações de regressão, foram verificadas antes da interpretação do modelo.

Avaliou-se a normalidade dos valores de KIM-1 utilizando o teste de *Shapiro–Wilk*. Diante de desvio significativo da normalidade, as análises foram repetidas utilizando valores de KIM-1 transformados em log. Os resultados foram consistentes com as análises primárias.

Devido aos tamanhos desiguais dos grupos, empregou-se o ajuste pela média harmônica na estimativa *post hoc*. Um valor de p bicaudal < 0,05 foi considerado estatisticamente significativo. Todas as análises foram realizadas utilizando o software SPSS versão 21.0.

### Considerações éticas

O estudo recebeu aprovação do Comitê de Ética em Pesquisa do Hospital Geral Dr. Soetomo (IRB nº 1197/KEPK/XII/2024). O consentimento informado por escrito foi obtido dos pais ou responsáveis legais de todos os participantes.

## RESULTADOS

### Características dos pacientes

Trinta pacientes pediátricos com diagnóstico recente de GNRP foram incluídos no estudo. A idade mediana foi de 15 anos (intervalo 3–17), com leve predominância do sexo feminino. Com base na classificação sorológica, 20 pacientes (67%) foram categorizados como GNRP associada a imunocomplexos, 6 (20%) como GNRP associada a ANCA (pauci-imune) e 4 (13%) como doença anti-GBM. As características basais demonstraram clara heterogeneidade entre os subtipos. Pacientes com doença anti-GBM apresentaram comprometimento renal mais grave, refletido por níveis mais elevados de creatinina sérica e maior necessidade de hemodiálise e de admissão em unidade de terapia intensiva (UTI) no momento do diagnóstico. Em contraste, a GNRP associada a imunocomplexos foi caracterizada por níveis mais baixos de complemento e maior frequência de positividade para ANA e anti-dsDNA, enquanto a GNRP associada a ANCA apresentou níveis de complemento preservados e distribuição dos subtipos de ANCA consistente com doença pauci-imune. Os valores basais de TFGe não diferiram significativamente entre os subtipos (ANOVA F(2,27) = 1,314; p = 0,285). Os níveis basais de KIM-1 sérica também não diferiram significativamente entre os subtipos (ANOVA F(2,27) = 1,631; p = 0,214).

Os valores são apresentados como média ± desvio padrão ou mediana (intervalo), conforme apropriado. A TFGe foi calculada utilizando a fórmula de *Schwartz atualizada*. As medições laboratoriais foram obtidas no momento do diagnóstico, antes do início da terapia de indução. HPF: campo de grande aumento (*high-power field*). As características basais demográficas, clínicas e laboratoriais, estratificadas conforme o subtipo de GNRP baseado em critérios sorológicos, estão apresentadas na Tabela 1.

**Tabela 1:** Características basais dos pacientes de acordo com o subtipo de GNRP baseado em sorologia

Variável	Imunocomplexos (n = 20)	Associada a ANCA (n = 6)	Anti-GBM (n = 4)
**A. Características demográficas**			
Idade, anos, mediana (intervalo)	14,5 (6–17)	14,5 (3–17)	15 (11–16)
Sexo feminino, n (%)	12 (60,0)	4 (66,7)	0 (0)
Índice de massa corporal, kg/m^2^, média ± DP	19,7 ± 0,6	20,2 ± 1,2	18,1 ± 3,3
**B. Características clínicas basais**			
Pressão arterial sistólica, mmHg, média ± DP	157,1 ± 3,4	153,7 ± 7,3	151,5 ± 8,3
Pressão arterial diastólica, mmHg, média ± DP	101,3 ± 1,2	102,0 ± 3,8	100,7 ± 4,6
Hemodiálise na apresentação, n (%)	11 (55,0)	0 (0)	3 (75,0)
Admissão em UTI, n (%)	10 (50,0)	0 (0)	3 (75,0)
**C. Função renal basal**			
Creatinina sérica, mg/dL, mediana (intervalo)	4,1 (1,8–30,4)	3,2 (1,8–4,5)	5,4 (3,2–8,2)
TFGe, mL/min/1,73 m^2^, média ± DP	20,4 ± 16,2	30,6 ± 5,4	17,2 ± 2,3
Nitrogênio ureico sanguíneo (BUN), mg/dL, média ± DP	102,0 ± 14,2	71,5 ± 19,7	81,7 ± 23,4
**D. Achados urinários**			
Eritrócitos urinários (por campo de grande aumento [HPF]), mediana (intervalo)	24 (13–348)	38 (11–89)	31 (19–488)
Relação albumina/creatinina, mg/gCr	≥300 (todos os pacientes)	≥300 (todos os pacientes)	≥300 (todos os pacientes)
**E. Biomarcadores específicos da doença**			
C3, mg/dL, média ± DP	53,4 ± 4,7	111,6 ± 6,5	103,2 ± 5,1
C4, mg/dL, média ± DP	17,1 ± 2,1	28,9 ± 5,2	28,9 ± 3,9
ANA positivo, n (%)	11 (55,0)	0 (0)	0 (0)
Anti-dsDNA positivo, n (%)	9 (45,0)	0 (0)	0 (0)
MPO-ANCA positivo, n (%)	0 (0)	4 (66,7)	0 (0)
PR3-ANCA positivo, n (%)	0 (0)	3 (50,0)	0 (0)
Título de anti-GBM, mediana (intervalo)	6,9 (1,1–17,3)	8,4 (1,6–18,3)	50,6 (42,7–142,5)
**F. Terapia de indução**			
Baseada em ciclofosfamida, n (%)	12 (60,0)	3 (50,0)	3 (75,0)
Baseada em MMF, n (%)	8 (40,0)	3 (50,0)	1 (25,0)

### Distribuição da terapia de indução

A distribuição da terapia de indução variou entre os subtipos. A terapia baseada em ciclofosfamida foi administrada com maior frequência em pacientes com doença anti-GBM e naqueles que apresentavam maior gravidade clínica, enquanto a terapia baseada em micofenolato mofetil (MMF) foi mais comumente utilizada na GNRP associada a imunocomplexos.

### Complicações relacionadas ao tratamento

Nenhuma infecção oportunista grave, necessidade de ventilação mecânica por causas infecciosas ou mortalidade ocorreu durante o período de indução de 3 meses. Infecções menores foram manejadas de forma conservadora, sem necessidade de interrupção da terapia imunossupressora.

### Alterações na TFGe

Todos os subtipos de GNRP baseados em sorologia apresen­taram melhora da TFGe após 3 meses de terapia de indução. Os valores médios de ΔTFGe foram numericamente mais elevados no grupo anti-GBM, seguidos pela GNRP associada a ANCA (pauci-imune) e pela GNRP associada a imuno­complexos. No entanto, a análise multivariada não demonstrou diferença estatisticamente significativa no ΔTFGe entre os três subtipos (MANOVA, p > 0,05).

Os detalhes da TFGe basal, da TFGe após 3 meses e do ΔTFGe para cada subtipo e para a coorte total são apresentados na Tabela 2. Na coorte total (n = 30), a TFGe média aumentou de 22,1 ± 15,1 para 77,3 ± 53,1 mL/min/1,73 m^2^, correspondendo a um ΔTFGe médio de +55,2 ± 44,8 mL/min/1,73 m^2^.

**Tabela 2:** Alterações na TFGe (ΔTFGe) após 3 meses de acordo com o subtipo de GNRP

Subtipo de GNRP	TFGe basal (média ± DP, mL/min/1,73 m^2^)	TFGe após 3 meses (média ± DP, mL/min/1,73 m^2^)	ΔTFGe (média ± DP, mL/min/1,73 m^2^)
Anti-GBM	17,2 ± 4,6	95,5 ± 79,9	78,2 ± 82,6
Imunocomplexos	20,4 ± 16,2	67,1 ± 52,1	46,7 ± 39,4
Associada a ANCA (pauci-imune)	30,6 ± 13,2	98,9 ± 31,9	68,3 ± 26,5
Coorte total	22,1 ± 15,1	77,3 ± 53,1	55,2 ± 44,8

Nota – Valores basais e após 3 meses da TFGe, bem como as alterações correspondentes (ΔTFGe) após terapia de indução, estratificados de acordo com o subtipo de GNRP baseado em sorologia. As diferenças entre os grupos foram avaliadas por meio de análise multivariada de variância (MANOVA).

A distribuição do ΔTFGe entre os subtipos é ilustrada na Figura 1.

**Figura 1:** Distribuição das alterações na taxa de filtração glomerular estimada (ΔTFGe) após 3 meses entre os subtipos de GNRP baseados em sorologia.

### Alterações na KIM-1 sérica

As concentrações séricas de KIM-1 diminuíram em todos os subtipos de GNRP baseados em sorologia ao longo do período de seguimento de 3 meses, de forma consistente com a melhora da lesão tubular. A maior redução numérica foi observada na GNRP anti-GBM, enquanto a menor ocorreu na GNRP associada a ANCA. No entanto, as diferenças no ΔKIM-1 entre os subtipos não foram estatisticamente significativas (p > 0,05). Em todos os grupos, os valores basais de KIM-1 sérica estavam marcadamente elevados em comparação com o intervalo de referência institucional (<20 pg/mL).

Os valores de KIM-1 sérica no momento basal e após 3 meses de terapia de indução, bem como as alterações correspondentes (ΔKIM-1), para cada subtipo de GNRP baseado em sorologia e para a coorte total, estão apresentados na Tabela 3.

**Tabela 3:** Alterações na KIM-1 sérica (ΔKIM-1) após 3 meses de acordo com o subtipo de GNRP

Subtipo de GNRP	KIM-1 basal (média ± DP, pg/mL)	KIM-1 após 3 meses (média ± DP, pg/mL)	ΔKIM-1 (média ± DP, pg/mL)
Anti-GBM	163,8 ± 157,5	80,7 ± 98,8	–83,1 ± 196,4
Imunocomplexos	138,9 ± 107,8	84,5 ± 81,9	–54,4 ± 125,3
Associada a ANCA (pauci-imune)	58,7 ± 31,5	52,2 ± 86,3	–6,5 ± 87,5
Coorte total	126,2 ± 107,6	77,5 ± 82,8	–48,6 ± 127,1

A distribuição de ΔKIM-1 entre os subtipos é ilustrada na Figura 2.

**Figura 2:** Distribuição das alterações na KIM-1 sérica (ΔKIM-1) após 3 meses entre os subtipos de GNRP baseados em sorologia.

### Resultados da ANCOVA

Para abordar possíveis efeitos de regressão à média, modelos de ANCOVA foram realizados utilizando os valores aos 3 meses como variáveis dependentes e os valores basais correspondentes como covariáveis. Após o ajuste, o subtipo baseado em sorologia não apresentou associação significativa com a TFGe aos 3 meses (p = 0,288). Como os valores de KIM-1 não apresentaram distribuição normal, modelos de ANCOVA com transformação logarítmica foram considerados primários para a análise de KIM-1. No modelo ajustado com transformação logarítmica, o subtipo permaneceu não significativo (F(2,26) = 0,202; p = 0,818; η^2^ parcial = 0,015).

Como os valores de KIM-1 apresentaram distribuição não normal (Shapiro–Wilk, p < 0,001), as análises de ANCOVA foram repetidas utilizando valores transformados em log. Após a transformação, o subtipo baseado em sorologia permaneceu não significativo (F(2,26) = 0,202; p = 0,818; η^2^ parcial = 0,015), confirmando a robustez dos achados. Não foi observada interação significativa entre os valores basais e o subtipo, o que sustenta a suposição de homogeneidade das inclinações de regressão. Os resultados da análise de covariância (ANCOVA), avaliando os desfechos em 3 meses após ajuste para os valores basais, estão apresentados de forma resumida na Tabela 4.

**Tabela 4:** Modelo de ANCOVA para TFGe aos 3 meses ajustado pela TFGe basal

Desfecho	F da covariável	p da covariável	F do subtipo	p do subtipo	R^2^
TFGe aos 3 meses	20,141	<0,001	1,307	0,288	0,479
KIM-1 aos 3 meses*	0,196	0,662	0,202	0,818	0,032

Nota – *Os valores de KIM-1 foram transformados em log para a análise de ANCOVA.

### Análise multivariada

A análise multivariada de variância combinando ΔTFGe e ΔKIM-1 não demonstrou efeito global significativo do subtipo de GNRP baseado em sorologia (Lambda de Wilks = 0,882; F(4,52) = 0,840; p = 0,506; η^2^ parcial = 0,061). Esses achados indicam que a classificação por subtipo não esteve associada a alterações combinadas em curto prazo nos marcadores glomerulares e tubulares. Considerando o pequeno tamanho dos subgrupos, particularmente no grupo anti-GBM (n = 4), o estudo pode não ter poder estatístico suficiente para detectar tamanhos de efeito moderados; portanto, resultados não significativos não devem ser interpretados como evidência de equivalência.

### Resumo dos principais achados

- O subtipo de GNRP baseado em sorologia não esteve associado à melhora em curto prazo da TFGe.
- O subtipo de GNRP baseado em sorologia não esteve associado à redução da KIM-1 sérica.
- As diferenças observadas entre os subtipos foram apenas numéricas e não alcançaram significância estatística.
- A recuperação renal precoce pareceu ser mais influenciada pela gravidade basal da doença e pela resposta terapêutica do que pela classificação do subtipo sorológico.

## DISCUSSÃO

Este estudo avaliou se os subtipos de GNRP pediátrica baseados em sorologia — nomeadamente, doença por anticorpos anti-membrana basal glomerular (anti-GBM), GNRP associada a imunocomplexos e GNRP associada a ANCA (pauci-imune) — estavam associados à recuperação renal em curto prazo, avaliada por meio das alterações na taxa de filtração glomerular estimada (ΔTFGe) e na molécula-1 de lesão renal sérica (ΔKIM-1) após terapia de indução. O principal achado deste estudo é que o subtipo de GNRP baseado em sorologia não esteve significativamente associado à melhora precoce da função glomerular nem aos marcadores de lesão tubular durante os primeiros 3 meses de tratamento. Esses achados ressaltam que, na GNRP pediátrica, a recuperação funcional precoce pode não refletir diretamente as diferenças etiológicas capturadas pela classificação sorológica durante a fase aguda do tratamento.

### Diversidade sorológica, mas padrões semelhantes de recuperação precoce

A GNRP representa uma síndrome clínica heterogênea caracterizada por rápida perda da função renal em um curto período de tempo, tradicionalmente categorizada de acordo com distintos mecanismos imunológicos^1^. A doença anti-GBM é mediada por autoanticorpos dirigidos contra a cadeia α3 do colágeno tipo IV, levando à lesão glomerular agressiva^2,3^. A GNRP associada a ANCA é caracterizada por glomerulonefrite necrosante com formação de crescentes e mínima deposição imune, frequentemente acompanhada de manifestações sistêmicas de vasculite^10,11^. Já a GNRP associada a imunocomplexos abrange um amplo espectro de doenças, incluindo glomerulonefrite pós-infecciosa, nefropatia por IgA e nefrite lúpica, que frequentemente apresentam evolução variável e potencialmente reversível em crianças^12^.

Apesar desses mecanismos biológicos iniciais distintos, nosso estudo não demonstrou diferenças estatisticamente significativas em ΔTFGe ou ΔKIM-1 entre os subtipos de GNRP baseados em sorologia durante a fase inicial do tratamento. Esse achado sugere que, no contexto agudo, a recuperação renal precoce na GNRP pediátrica pode ser mais determinada por vias inflamatórias descendentes compartilhadas e pela resposta à terapia imunossupressora do que pelo processo sorológico desencadeador em si. Tal convergência das trajetórias de recuperação precoce pode obscurecer diferenças específicas entre subtipos que, possivelmente, se tornariam evidentes apenas com seguimento mais prolongado.

É plausível que, apesar de diferentes gatilhos imunológicos iniciais, a via final comum de formação de crescentes e inflamação glomerular aguda convirja para trajetórias funcionais semelhantes no curto prazo após o início da terapia imunossupressora. Essa convergência biológica pode atenuar as diferenças precoces entre subtipos na função renal mensurável.

### Interpretação dos achados de TFGe

Embora a GNRP anti-GBM tenha apresentado o maior aumento numérico na TFGe, seguida pela GNRP associada a ANCA e pela GNRP associada a imunocomplexos, essas diferenças não alcançaram significância estatística. Diversos fatores podem explicar essa observação. Primeiro, a terapia de indução na GNRP é projetada para suprimir rapidamente a inflamação glomerular independentemente da etiologia, o que pode minimizar diferenças precoces na recuperação funcional entre os subtipos^1^. Segundo, a gravidade basal da doença pareceu influenciar o ΔTFGe; pacientes que se apresentam com comprometimento renal mais grave podem demonstrar maiores melhorias numéricas devido a uma maior margem para recuperação, sem necessariamente alcançar melhores desfechos em longo prazo^2^. Terceiro, a distribuição desigual de pacientes entre os subtipos e o tamanho limitado da amostra reduziram o poder estatístico para detectar diferenças modestas entre os grupos.

Um seguimento de 3 meses capta principalmente a resposta inflamatória inicial, em vez do remodelamento estrutural de longo prazo. Diferenças relacionadas ao subtipo na progressão da fibrose, no risco de recaída ou no desenvolvimento de doença renal crônica podem emergir apenas após períodos de observação mais prolongados^3^.

### Interpretação dos achados de KIM-1

A KIM-1 é um biomarcador bem estabelecido de lesão tubular proximal, marcadamente superexpresso após dano epitelial e representativo tanto de estresse tubular agudo quanto crônico^13,14^. No presente estudo, os níveis séricos de KIM-1 diminuíram em todos os subtipos de GNRP baseados em sorologia após a terapia de indução, de forma consistente com a melhora da lesão tubular. No entanto, a magnitude de ΔKIM-1 não diferiu significativamente entre os subtipos.

Esse achado reflete provavelmente a cinética mais lenta e heterogênea da reparação tubular em comparação com a recuperação funcional glomerular. A regeneração epitelial tubular frequentemente ocorre de forma mais tardia em relação às melhorias na filtração e, portanto, as alterações precoces em KIM-1 podem ser menos sensíveis às diferenças etiológicas na fase aguda^13^. Além disso, a substancial variabilidade interindividual nos níveis basais de KIM-1 e nas suas alterações ao longo do tempo limita ainda mais a capacidade discriminatória da dinâmica de KIM-1 em curto prazo entre os subtipos sorológicos. Essas observações sugerem que um seguimento mais prolongado pode ser necessário para determinar se os biomarcadores tubulares podem diferenciar de forma significativa as trajetórias de recuperação na GNRP pediátrica.

Como análises baseadas em Δ podem ser influenciadas pela gravidade basal, modelos de ANCOVA foram realizados para ajustar os valores basais. Mesmo após o controle da TFGe e da KIM-1 basais, o subtipo baseado em sorologia não esteve associado à recuperação renal em curto prazo. Esses achados reforçam a robustez dos resultados principais e sugerem que a melhora funcional precoce pode ser independente da classificação sorológica.

Embora a KIM-1 urinária seja mais comumente utilizada como biomarcador de lesão tubular proximal, neste estudo foi realizada a medição da KIM-1 sérica por razões práticas e de viabilidade em um contexto pediátrico agudo. A coleta de urina pode ser pouco confiável em pacientes criticamente enfermos, particularmente naqueles submetidos à hemodiálise. No entanto, a KIM-1 sérica pode ser influenciada por inflamação sistêmica, redução da depuração renal e dinâmica das proteínas circulantes, o que pode limitar sua especificidade em comparação com as medições urinárias. Isso representa uma limitação importante na interpretação da dinâmica da lesão tubular em nossa coorte.

### Implicações clínicas

Os achados deste estudo têm importantes implicações clínicas. Embora a classificação sorológica permaneça essencial para a orientação diagnóstica e para a tomada de decisões terapêuticas na GNRP pediátrica, ela não deve ser utilizada isoladamente para predizer a recuperação renal em curto prazo. A ausência de diferenças significativas nas alterações precoces da TFGe e da KIM-1 entre os subtipos de GNRP baseados em sorologia indica que o prognóstico inicial não depende do subtipo. Em vez disso, o diagnóstico oportuno e o início precoce da terapia imunossupressora apropriada provavelmente desempenham um papel mais decisivo na determinação dos desfechos precoces do que o subtipo sorológico isoladamente^6^. Do ponto de vista clínico, esses achados sugerem que as estratégias de manejo inicial na GNRP pediátrica devem priorizar a gravidade da doença e o início oportuno da terapia imunossupressora, em vez de depender exclusivamente do subtipo sorológico para a predição prognóstica em curto prazo.

### Pontos fortes, limitações e direções futuras

O tamanho relativamente pequeno da amostra, particularmente nos subgrupos anti-GBM (n = 4) e associados a ANCA (n = 6), limita substancialmente o poder estatístico e aumenta o risco de erro do tipo II. Portanto, a ausência de diferenças estatisticamente significativas não deve ser interpretada como evidência de equivalência entre os subtipos de GNRP baseados em sorologia. Considerando a raridade da GNRP pediátrica, este estudo deve ser considerado exploratório, e coortes multicêntricas maiores são necessárias para confirmar esses achados. Até onde sabemos, este é um dos poucos estudos prospectivos pediátricos que avaliam simultaneamente a função glomerular e os marcadores de lesão tubular na fase inicial da GNRP. A inclusão de modelos de ANCOVA e análises com transformação logarítmica reforça a robustez dos achados, apesar do tamanho limitado da amostra.

No entanto, várias limitações devem ser reconhecidas. O tamanho reduzido da amostra, particularmente nos grupos anti-GBM e associados a ANCA, limitou o poder estatístico. O período relativamente curto de seguimento pode não captar os desfechos renais em longo prazo ou a progressão para doença renal crônica. Como a classificação dos subtipos foi baseada exclusivamente em critérios sorológicos, não se pode excluir a possibilidade de viés de classificação incorreta, particularmente no grupo associado a imunocomplexos, que pode incluir entidades heterogêneas, como nefrite lúpica ou glomerulonefrite relacionada à infecção. A confirmação histopatológica poderia ter permitido a avaliação da porcentagem de crescentes, do índice de cronicidade e da fibrose túbulo-intersticial, que são preditores conhecidos de desfechos renais. A ausência desses parâmetros estruturais limita nossa capacidade de correlacionar o subtipo biológico com a gravidade da lesão em nível tecidual^15^. Estudos futuros devem envolver coortes pediátricas multicêntricas maiores e com períodos de seguimento mais longos para melhor delinear as trajetórias de recuperação específicas de cada subtipo. A integração de biomarcadores tubulares adicionais e de avaliações estruturais poderá esclarecer ainda mais a relação entre o fenótipo sorológico, a lesão tecidual e os desfechos renais em longo prazo^16^. Em conclusão, nossos achados sugerem que a recuperação funcional renal precoce na GNRP pediátrica pode refletir dinâmicas inflamatórias compartilhadas, em vez de depender exclusivamente da categorização sorológica, ressaltando a importância da intervenção terapêutica oportuna em todos os subtipos.

## Data Availability

Os dados estão disponíveis com o autor correspondente mediante solicitação razoável, com a garantia de anonimato dos pacientes.
